# The Cytoskeleton of the Retinal Pigment Epithelium: from Normal Aging to Age-Related Macular Degeneration

**DOI:** 10.3390/ijms20143578

**Published:** 2019-07-22

**Authors:** Ioana-Sandra Tarau, Andreas Berlin, Christine A. Curcio, Thomas Ach

**Affiliations:** 1 Department of Ophthalmology, University Hospital Würzburg, 97080 Würzburg, Germany; 2 Department of Ophthalmology and Visual Sciences, University of Alabama at Birmingham, Birmingham, AL 35249, USA

**Keywords:** retinal pigment epithelium, cytoskeleton, aging, age-related macular degeneration, actin, microfilament, microtubules, stress fiber

## Abstract

The retinal pigment epithelium (RPE) is a unique epithelium, with major roles which are essential in the visual cycle and homeostasis of the outer retina. The RPE is a monolayer of polygonal and pigmented cells strategically placed between the neuroretina and Bruch membrane, adjacent to the fenestrated capillaries of the choriocapillaris. It shows strong apical (towards photoreceptors) to basal/basolateral (towards Bruch membrane) polarization. Multiple functions are bound to a complex structure of highly organized and polarized intracellular components: the cytoskeleton. A strong connection between the intracellular cytoskeleton and extracellular matrix is indispensable to maintaining the function of the RPE and thus, the photoreceptors. Impairments of these intracellular structures and the regular architecture they maintain often result in a disrupted cytoskeleton, which can be found in many retinal diseases, including age-related macular degeneration (AMD). This review article will give an overview of current knowledge on the molecules and proteins involved in cytoskeleton formation in cells, including RPE and how the cytoskeleton is affected under stress conditions—especially in AMD.

## 1. Introduction

### The Retinal Pigment Epithelium

The retinal pigment epithelium (RPE) is a cellular monolayer located between the photoreceptor outer segments (apical) and the choroidal vasculature (basal). The RPE’s own basal lamina is historically considered to be one of the five layers of the Bruch membrane—the inner wall of the choroid [1]. The RPE has several functions including the transportation of ions, water and metabolic products from the subretinal space to the blood vessels of the choriocapillaris [2]; on the other hand, it also takes up nutritional and metabolic essentials such as fatty acids, retinol and glucose from choriocapillaris and delivers these products to the photoreceptors [3]. It plays a central role in the visual cycle, i.e., re-isomerization from all-trans-retinal to 11-cis-retinal [4]. Another major RPE function is the daily phagocytosis of the shed photoreceptor outer-segment tips and intracellular processing and recycling of their constituents [5,6].

A hallmark of this monolayer is a strong apical to basal polarization, which enables the controlled trafficking of proteins and molecules, nutrients, oxygen, and waste products from and into RPE cells [7,8]. This has been shown to be important for the RPE cellular metabolism, as well as maintaining outer retina health. RPE cells are able to release molecules and proteins or exocytose material into the sub-RPE space (on the basal aspect, between the basal membrane of the RPE cell and the inner collagenous layer of the Bruch membrane) or into the inter-photoreceptor matrix of the subretinal space (between apical processes of the RPE cells and outer segments of the photoreceptors) [9,10,11,12,13,14]. 

Intracellularly, RPE cells accumulate different types of organelles (granules) that are relevant in clinical retinal imaging: lipofuscin and melanolipofuscin granules, as well as melanosomes [15,16]. Lipofuscin and melanolipofuscin have autofluorescent features and emit light after blue light excitation. This property has widely been used for in vivo imaging of ocular fundus autofluorescence for more than two decades [17,18]. In addition, intracellular mitochondria exhibit a high reflectivity, which is clinically visible in spectral domain optical coherence tomography (SD-OCT) [19,20].

The macula of the human retina has sub-regions of distinctive photoreceptor composition (all-cone fovea, rod-dominant perifovea) that are expected to impact the structure and function of the underlying RPE [21]. Although a monolayer, the RPE cells of human retina show distinct phenotype differences in relation to their position relative to the fovea—RPE cells at the fovea are smaller in diameter, while intracellular lipofuscin granule deposition favors the perifovea [22]. Also, at the perifovea, the highest number of multinucleated RPE cells exist, while multinucleated RPE cells are nearly absent at the fovea [23]. These differences might represent an adaptation to different needs in direct connection and communication with the overlying photoreceptors, supporting the concept that RPE cells and the overlying photoreceptors form a functional unit to sample visual space [21]. A pre-requisite for RPE polarization is the watertight outer blood–retinal barrier formed by a circumferential belt of RPE junctional complexes [8]. Some important intra- and extracellular structures in maintaining this barrier, and the RPE cell shape and morphology, are proteins and multimolecular complexes directly on RPE cells (tight junction, zonula adherens, desmosomes), within RPE cells (membrane cytoskeleton, membrane proteins), or external to the RPE (extracellular matrix) [24,25,26].

The purpose of this review is to summarize the composition, cellular localization, and function of RPE cytoskeletal constituents, with a special focus on changes in normal aging and in age-related macular degeneration (AMD). 

## 2. Cytoskeleton of the Retinal Pigment Epithelium

**Figure 1:**
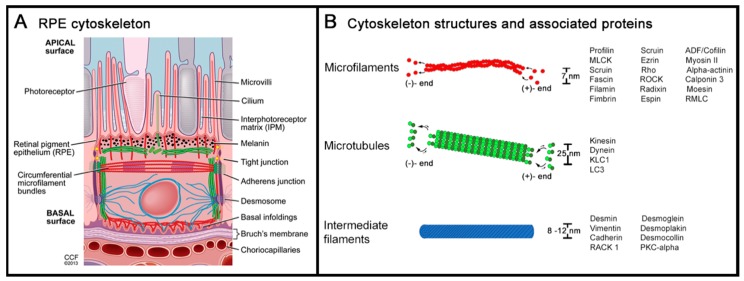
The cytoskeleton of the retinal pigment epithelium (RPE). (**A**) A cross-sectional view of the RPE and the adjacent photoreceptor outer segments (apical) and Bruch membrane (basal) highlights the intracellular network of cytoskeleton proteins: actin filaments, microtubules, and intermediate filaments. (**B**) The graph plots the dimension and protein structure of the above-mentioned cytoskeleton members: microfilaments consist of helical arranged polymers of actin proteins with an ATP-rich assembling (+)-end and a less energetic, ADP-rich disassembling (-)-end. Microtubules are hollow cylinders of tubulin proteins, also possessing an energetic, GTP-rich assembling (+)-end and a less energetic GDP-rich disassembling (-)-end. Intermediate filaments form rope-like fibers consisting of a large group of filament-organizing proteins. The regulating and associated proteins of each of the cytoskeleton substructures as mentioned in this review are listed (for details and additional regulating proteins, see detailed reviews from Bonilha and Hohmann et al. [32,33]). Figure 1A: a reprint with permission from Elsevier (via RightsLink) and Vera Bonilha, PhD, and the Cleveland Clinic Center for Medical Art & Photography [32].

The cytoskeleton is one of the most multifaceted and complex structures in biological tissues. In addition to providing a structural scaffold for the cell, it also actively participates in a range of processes such as endocytosis, cell division, intracellular transport, adhesion, motility, force transmission, responding to external forces, and adapting cell shape and cell layer geometry to external and internal factors [27,28,29]. RPE cells exhibit most of these functions, which emphasizes the importance of the cytoskeleton in maintaining outer retinal homeostasis. As with other cell types, the RPE cytoskeleton (Figure 1A) is comprised of three classic filament types: actin, microtubules, and intermediate filaments (Figure 1B) [30,31,32]. By forming a distinctively structured, dynamic, and communicating network, the RPE cytoskeleton reacts and adapts to signals—both intra- or extracellularly—by a continuing re-organization of the network. 

In the following paragraphs, the major cytoskeleton proteins are explained in more detail. It is worth mentioning here that the knowledge of cytoskeleton composition, involved proteins, and changes in pathological conditions is based on findings in both RPE and mostly non-RPE cells. This is acknowledged whenever appropriate. 

### 2.1. Actin and Non-Muscle Myosin 

Actin is a highly dynamic cytoskeletal protein, with a strong ability to adapt to structural changes, thus determining the shape of a cell. Actin microfilaments form a crosslinked grid network of polarized filaments supporting intracellular vesicle trafficking. These microfilaments also interact with several connecting proteins both intracellularly and at the cell border, providing support for focal adhesion, cell shape changes and cell movement [34]. Actin emerges as a branching network with viscoelastic behavior: mainly elastic on shorter time scales (<1 min) and more viscous on longer time scales (>10 min) [35,36]. Actin occurs either as monomeric G-actin or filamentous F-actin. In an ATP-dependent reaction, polarized G-actin polymerizes to asymmetric helical structures (= the polarized filamentous actin (F-actin) [37]) with a less dynamic (-)-end and the more dynamic (+)-end [38,39,40,41]. Every actin subunit is able to bind ATP and to hydrolyze it to ADP for filament growth, occurring at the (+)-end [42]. 

Actin polymerization is controlled by several cofactor-driven elements. Profilin, an actin-binding protein, catalyzes the transition from ADP- to ATP-actin and regulates actin formation [43,44]. Also, actin closely interacts with the motor-protein myosin [45]. Myosin affects both the retraction of a cell during movement and also transmits forces to the extracellular matrix. However, forces on actin filaments are feasible only when myosin II hexamers have a bipolar filament appearance [46,47]. Myosin activation is regulated by several kinases (inter alia Rho-associated protein kinase, myosin light chain kinases) and phosphorylation of the regulatory light chain or phosphorylation of the myosin heavy chain [48,49,50,51,52,53,54]. 

Myosin belongs to a group of intracellular cross-linker proteins, which connect single actin filaments either transiently/non-transiently and/or passively (e.g., via scruin, fascin, α-actinin, filamin, or fimbrin) or actively (myosin). Using ATP as an energy supply, myosin is responsible for the contractility of actin structures [55,56]. The interaction of actin with its cross-linkers largely controls shape, mechanical integrity, and contractility of cells [29,57,58]. 

Most actin structures are stable over time. However, to adapt to external environmental factors, cells need a mechanism to induce actin disassembly and re-organization. Therefore, the actin depolymerizing factor (ADF)/cofilin protein family binds to actin and is capable of disassembling and fragmenting actin filaments. ADF/cofilin does not alter the polymerization rate [59,60]. The efficiency of ADF/cofilin fragmentation highly depends on its binding abundance to actin filaments. Filaments that are fully covered with ADF/cofilin molecules are stabilized robustly, while actin filaments that are only partially occupied fragment faster [43,61,62]. Destabilization and fragmentation of the F-actin cytoskeleton is an observable finding in AMD-affected RPE cells (see below).

### 2.2. Microtubules and Intermediate Filaments

Microtubules consist of polymers originating from α/β-tubulin heterodimers. These tubular-shaped protein complexes have two different polymerization ends (+ and -). Microtubules play an important role in mitosis, cellular growth, cell shape control, and active intracellular transport [63,64,65] (through kinesin and dynein proteins), facilitated by the microtubule’s polarity. GTP-dependent polymerization and depolymerization is also important during mitosis. 

In RPE cells, microtubules are involved in the phagocytosis of photoreceptor outer segment tips. Phagosomes use the microtubule network as a guide rail for intracellular travel: maintained by kinesin (plus-end directed), dynein (minus-end directed), and associated proteins (e.g., kinesin-1 light chain 1 (KLC1)). In mice, KLC1 deficiency impairs phagosome translocation in RPE cells and results in AMD-like pathologies [66] including thick basal laminar deposits and deposition of activated complement component C3b. Moreover, the microtubule-associated protein 1 light chain 3 (LC3) family of proteins is essential for maintaining normal lipid homeostasis in RPE-cells. Dhingra et al. recently showed that the systemic deletion of LC3B in mice resulted in an increased phagosome accumulation, a reduced fatty acid oxidation, and intracellular RPE lipid deposition [67]. Furthermore, as demonstrated in non-RPE cells, oxidative stress (also a major contributor to RPE cell disease) affects microtubules and can lead to cytoskeleton re-arrangement [68].

Intermediate filaments are 10 nm in diameter, i.e., they are intermediate in size between microtubules at 24 nm and microfilaments at 7 nm. Intermediate filaments are found in the nucleus and cytoplasm and consist of several proteins (neurofilaments, keratins, desmin, vimentin) [69]. This robust intracellular dynamic network is connected to cell-adhesive structures such as desmosomes at cell–cell contacts and hemi-desmosomes at cell–cell or cell–extracellular matrix contacts. The loss of these contacts leads to a loss of adhesion forces between the cells. Intracellularly linked to the actin and microtubule networks, intermediate filaments are responsible for signal transduction from the extracellular to the intracellular space [70] and participate in cell division and cell migration. Intermediate filaments are highly flexible and can be considered “shock absorbers,” since they are capable of massive elongation or compression without irreversible damage [71,72,73,74]. 

Under physiological conditions, the intermediate filaments keratin, vimentin, and desmin interact with desmoplakin, a cytolinker associated with desmosomal proteins such as cadherin, desmoglein and desmocollin [69]. Vimentin [75] and actin (among other proteins) are connected to plectin. This interaction decreases mitochondrial motility and elevates the mitochondrial membrane potential. Under stress conditions, phosphorylation of vimentin impacts the vimentin-mitochondria complex and leads to a disassembly of intermediate filaments, with increased motility and reduced ATP production by mitochondria [76,77].

Endocytosis is mediated by the interaction of keratin with the receptor for activated c kinase 1 (RACK1), which controls protein kinase C α (PKC α) activity [78]. The alteration of keratin filaments affects endocytosis and impacts intercellular vesicle transport. In addition, an intact keratin network is essential for polarized membrane traffic [79]. All forms of chemical, physical, or mechanical stress impact intermediate filaments and lead to an increased expression of these proteins [80,81,82,83]. It is worth mentioning that the RPE´s cytoskeleton comprises of keratins (among others, cytokeratin 5, 7, 8, 18, 19), with cytokeratin 18 being specific for RPE cells [84]. Cytokeratin 18 can be used as a marker protein in immunostaining and differentiating RPE.

### 2.3. Cytoskeleton at the Apical RPE

Microvilli at the apical surface of RPE cells increase the surface area and closely connect to the photoreceptor’s outer segments. Together with microtubules, tightly packed actin filaments within the microvilli help guarantee the structure, polarization, and orientation of apical processes [85,86]. Several molecules are important for a normal villi buildup: ezrin, a regulator of actin filaments, and actin-binding proteins such as villin, fimbrin and espin [87,88]. 

The daily phagocytosis of shed rod and cone photoreceptor outer segment tips requires flexibility, re-arrangement, and the correct orientation of the cytoskeleton [89]. Early studies found that uptake and intracellular processing of extracellular material is mediated by both actin and myosin, as well as microtubules for the fusion of lysosomes and phagosomes [90,91,92,93]. Alterations of the actin cytoskeleton and the microtubule system can have a major impact on outer retina health status, leading to a defect in photoreceptor outer-segment uptake and abnormal phagocytosis of the distal membrane stack [94] (of oldest membranes). 

The intracellular network of actin and microtubules is also considered important in the transport of pigment granules (melanosomes). Pigment granules within RPE cells move bi-directionally between the apical portion of the cell body and the apical processes [95,96]. Numerous melanosomes are found within the apical processes of RPE cells, as first discovered by single-section electron microscopy [97] and recently, quantified by volume electron microscopy [16]. The loss of melanosomes in the apical processes, or the inability of melanosomes to travel into the apical processes, is associated with severe degeneration of RPE cells and the outer retina [98]. 

Studies localized ezrin [87] and ERM (ezrin, radixin, moesin)-binding phosphoprotein 50 (EBP50) along entire actin filaments of microvilli, implicating this complex as responsible for the dynamics and regulation of microvilli components [99,100,101]. EBP 50, in direct interaction with the intracellular retinaldehyde binding protein (IRBP) [102,103,104,105], is responsible for the transport of hydrophobic retinoids between the RPE and photoreceptors. It also plays a crucial role in retinoid processing and in the visual cycle. The IRBP protein is coded by the gene *RBP3* (retinol-binding protein 3, on chromosome 10 in humans [106]). Mutations in or the absence of this gene are associated with retinal diseases including retinitis pigmentosa [107] and retinitis punctata albescens.

### 2.4. Cytoskeleton at the Basolateral RPE

At the basolateral site, the actin filament network is linked to zonula adherens and is responsible for cell-shape. In conjunction with filamin (a binding protein), myosin, tropomyosin (a structural protein), and α-actinin (an anchorage protein), actin filaments are part of circumferential microfilament bundles (CMB) which are contractile [108]. In the non-contractile state, tropomyosin and actin have no solid connection. Gunning et al. described a “floating” of the tropomyosin polymer over the surface of the actin filaments, as van der Waals interactions are missing [109]. However, due to mechanical or oxidative stress, these two components become tightly linked [110]. The phosphorylation of regulatory myosin light chain (RMLC) stimulates myosin activity and leads to contractile forces [111], which in turn, are a pre-requisite for the assembly of stress fibers. 

The exact molecular pathway of stress fiber formation remains unclear; however, numerous proteins such as calponin 3, Rho-associated coiled-coil kinases (ROCK) and RMLC that maintain stress fiber formation, disassembly, and contractility have been described [112,113,114]. In general, stress fibers are composed of bundles of multiple actin filaments [115] crosslinked by α-actinin [116]. They are connected to intermediate filaments and focal adhesions for signal transduction from the extracellular matrix (ECM) to the intracellular actin cytoskeleton [117,118,119]. 

Actin and myosin are two principal constituents of contractile stress fibers and the main contributors to cell contractility in many animal cells [119]. Non-contractile cells do not contain myosin [120]. Stress fibers have been well investigated in motile cells, where they can be divided into different categories based on their intracellular location, orientation, and contractility: the perinuclear actin cap, transverse (forming an arc across the top of a cell opposite from its base on a substrate), dorsal (connecting transverse and ventral fibers), and ventral stress fibers (connecting to focal adhesions of the cells to a substrate). Contractility involves the ventral and transverse stress fibers as well as the perinuclear actin cap. Myosin is present along these fibers. Stress fibers also contain actin (which binds proteins and focal adhesion-associated proteins [120,121,122,123]), as well as cross-linkers such as α-actinin [116], which stabilize the bundle and function as a signaling mediator. Transversal stress fibers form when the dendritic (branching) network collapses and is restructured by myosin [124,125].Ventral stress fibers form from existing dorsal stress fibers, as well as their fusion with attached transverse stress fibers [120,126]. The presence of dorsal stress fibers seems to be important for the assembly of the other stress fiber types and a link to focal adhesions [120,121]. Contractile stress fiber types are highly dependent on the presence and activity of myosin. As a result, myosin inhibition leads to the malfunction and degradation of stress fibers [127].

The RPE in its native state has few stress fibers. However, as AMD progresses and cells become dysmorphic, non-epithelioid, and even migratory, the processes described above for motile cells likely become operative [128]. Extracellular deposits (e.g., drusen in AMD, see below) can lead to mechanical stress within RPE cells. Mechanical stress (mechanosensing) is likely not the only trigger for stress fiber formation: thermic and chemical stress have also been discussed. 

### 2.5. Extracellular Matrix (ECM)

As mentioned earlier, RPE cells contact and interact with ECM via receptors such as integrins and cadherins [129,130]. The basal lamina of RPE cells has historically been considered as part of the 5-layered Bruch membrane (i.e., the basal lamina of the RPE cell, the inner and outer collagenous layer, the elastic fiber band, and the basement membrane of the choriocapillaris). It consists of collagen type IV, fibronectin, hyaluronic acid, and heparan and chondroitin sulfate [131]. Recently, the use of longitudinal eye-tracked clinical spectral domain optical coherence tomography (SD-OCT) has made it possible to appreciate the independent contributions of the RPE basal lamina to AMD progression, as it thickens into the basal laminar deposit [132,133]. Thus, an early suggestion that the Bruch membrane is better thought of as a three-layered structure (inner and outer collagenous layers, elastic layer) [134] is garnering a renewed interest [135].

The ECM is a dynamic structure with the ability to adapt to changes in the environment and is also important for cell adhesion and mechanical stability. The ECM also influences the polarity of the adjacent RPE [7]. ECM-RPE cell interactions play a key role in RPE cell adhesion to the Bruch membrane, as well as RPE cell differentiation, morphology, migration, and proliferation [136]. Furthermore, integrins seem to play an important role in the development of neovascular AMD, since altered integrin expression affects the RPE–ECM interaction [137]. Several RPE cell culture systems showed that an altered ECM also affects whole RPE cells, an important finding for the understanding of AMD changes including deposits both in the subretinal space (subretinal drusenoid deposits) and the sub-RPE space (classical soft/hard drusen; see below). 

### 2.6. RPE Geometry in Normal Aging 

Some of the above-mentioned cytoskeleton properties are necessary for meiosis and cell division. The RPE, however, is believed to be a non-dividing cell monolayer, with little or no signs of cell cycle activity under normal conditions [138]. Therefore, cytoskeleton responsibilities in RPE cells might be different from other cell types. 

A seminal 1978 paper by Wing, Blanchard, and Weiter demonstrated the exquisite correlation of lipofuscin-attributable autofluorescence with the topography of photoreceptors in human retina, which was known at the time [139,140]. The correlation of foveal cone and RPE topography was shown in horizontal tissue sections by Gao and Hollyfield [141]. Using flatmounts of human retina to produce digitally aligned maps of photoreceptors and the RPE [21,22], we demonstrated both a peak in RPE cell numbers under the fovea and the ring of high autofluorescence related to the ring of high rod density in the perifovea and near-periphery. These two studies with accurate foveal centration showed a stability of foveal RPE cell density with aging [22,141]. Ach et al. additionally showed a stability of extrafoveal RPE cell numbers despite an increase of autofluorescence [22]. 

**Figure 2:**
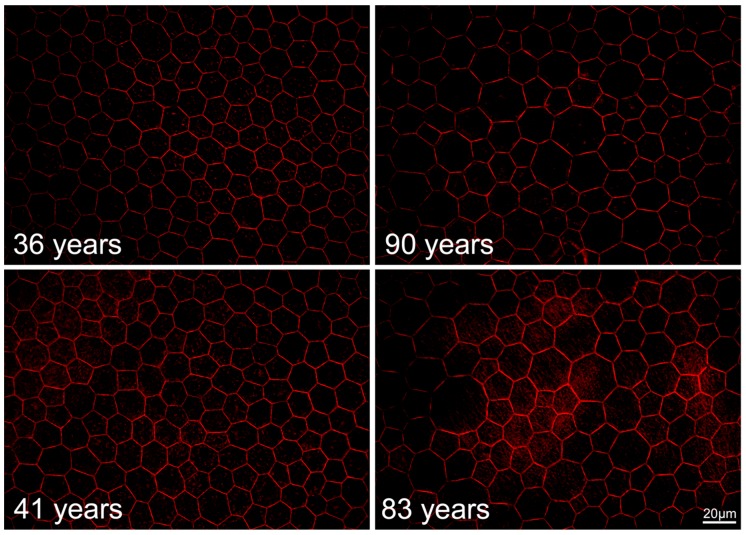
Age-related differences in the RPE cytoskeleton in normal eyes. In young humans (36, 41 years), a uniform geometry of polygonal, mostly hexagonal RPE cells is present, both at the fovea and near periphery. At older ages (83, 90 years) the strict geometry loosens and shows some enlarged cells. This leads to an altered arrangement of the RPE cells. However, orderly packing and a stringent geometry can still be recognized, even at an advanced age. Despite these subtle changes in geometry, the total number of RPE cells at the posterior pole remains stable [22,141,142]. The use of human tissue has previously been approved by the Institutional Review Board at University of Alabama at Birmingham, AL, USA (Protocol X900525013; September 11, 2012.

In normal aging, the RPE monolayer undergoes apparent changes, as recently shown in histological studies of human RPE flatmounts. In persons under 51 years, >90% of all RPE cells at the fovea are polygonal with sharp vertices and between 5 and 7 neighbors (59% hexagonal). The proportion of hexagonal cells decreases with increasing distance from the fovea. In advanced age (>80 years), these numbers slightly change with a reduction of hexagonal cells (52% at the fovea), resulting in a more irregular geometry (Figure 2). Interestingly, despite this change in geometry, the overall number of cells remains stable.

It is obvious that the RPE cytoskeleton is involved in this re-modeling of the cell layer and that structural changes in the ECM and the surrounding environment might be an initiator for cell layer responses. Age-related changes in the Bruch membrane (including the cross-linking of collagens, the deposition of lipids, increases in thickness, and reductions in elasticity and permeability), as well as basal laminar and basal linear deposits (lipids accumulated between the RPE basal lamina and inner collagenous layer of the Bruch membrane), might chemically and mechanically impact the RPE’s function and structure. 

### 2.7. RPE Cytoskeleton in AMD

**Figure 3:**
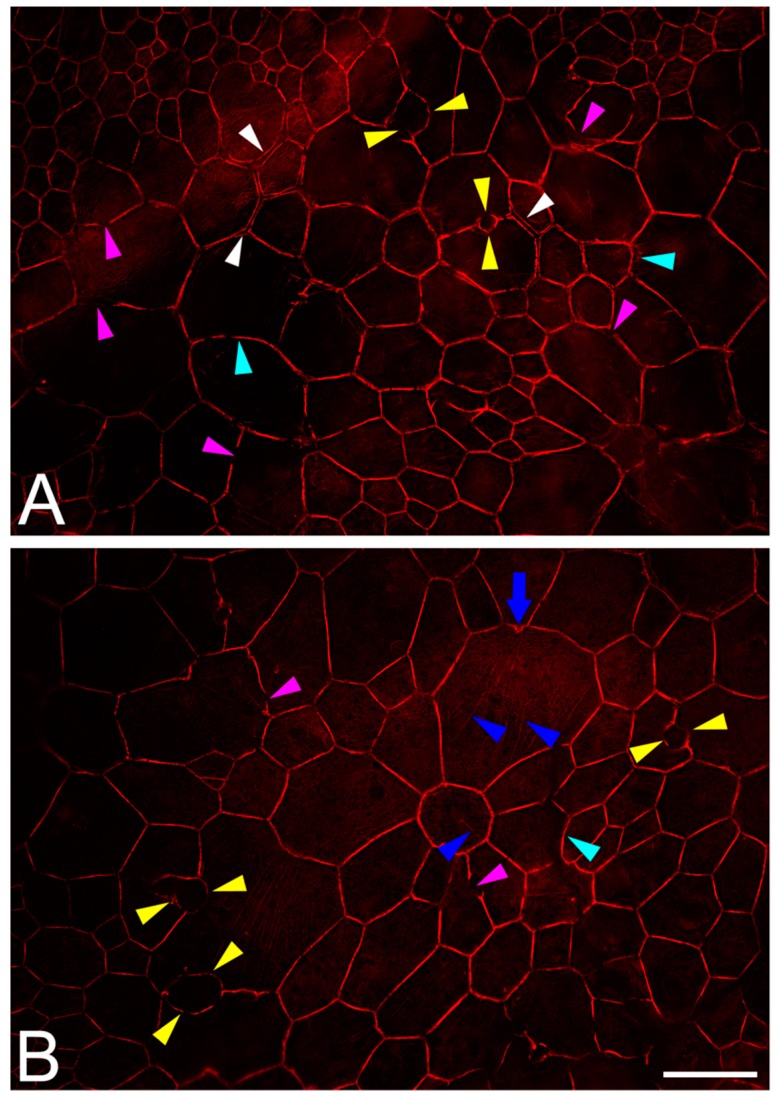
RPE cytoskeleton alterations in age-related macular degeneration (AMD). In contrast to the normal architecture and to age-related changes in RPE geometry (Figure 1), patchy areas with loss of the cells’ regular polygonal geometry and shape arise at AMD lesions (**A**,**B**). Prima facie, especially enlarged cells with a partly roundish shape (turquoise arrowheads) and variable irregular phenotypes are present. The ‘railroad tracks’-like arrangement of adjacent cells´ cytoskeleton bands is altered, showing separation (white arrowheads) and fragmentation or interruption (pink arrowheads). A special separation is sporadically seen: a splitting of the cytoskeleton (yellow arrowheads), which starts as small roundish lesions which then enlarge and finally might lead to a complete separation of two cells. A common finding in altered RPE cells is the presence of intracellular stress fibers (in **B**, blue arrowheads). At the insertion sites of the stress fibers, the actin filament cytoskeleton appears frayed and thickened (blue arrow). Donor: 83 years, female. RPE cells from the parafovea. F-actin labeled with AlexaFluor647-Phalloidin. Scale bar: 50 µm.

**Figure 4:**
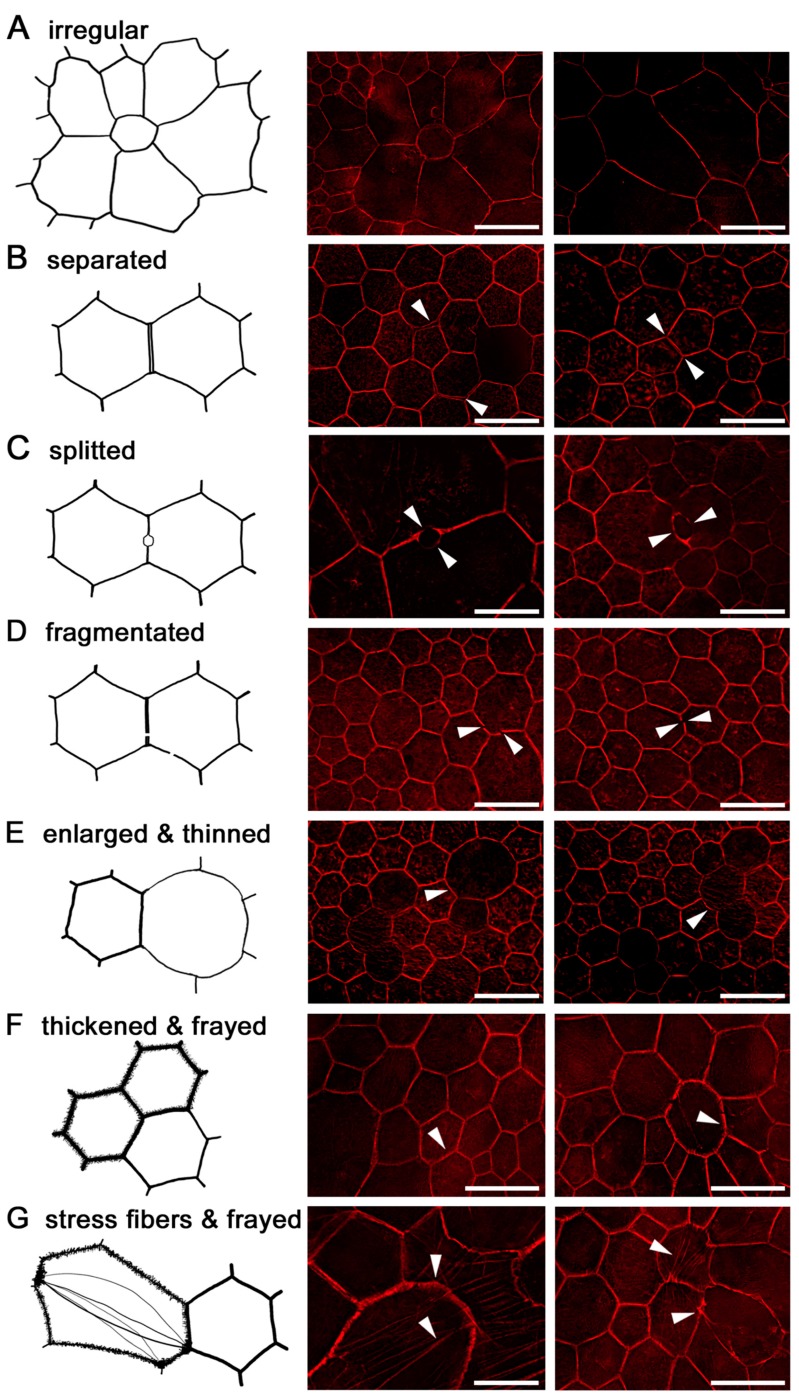
Cytoskeleton changes in AMD-affected eyes. In AMD, RPE cells and the cytoskeleton undergo significant changes. Drawings and photomicrographs show different cells with similar characteristics. (**A**) The loss of the cytoskeletons’ regular polygonal geometry and shape: enlarged cells with partly roundish cell shape and very variable cell sizes. (**B**) The separation of adjacent cells’ cytoskeleton bands. (**C**) The focal splitting of the cytoskeleton of two adjacent cells. These alterations are tiny in the beginning, but progress over time and can lead to a complete separation of two RPE cells. (**D**) Cytoskeleton fragmentation with dislocated free ends. Noticeably, the cell shape of these cells appears to be only minimally affected. (**E**) Enlarged RPE cells with a partly or complete thinned F-actin. (**F**) A thickened and frayed F-Actin. (**G**) In affected RPE cells, multiple intracellular stress fibers may appear. At sites where stress fibers insert, the cytoskeleton also appears frayed and thickened. Scale bar: 20 µm.

In addition to the previously mentioned age-related changes, the RPE cytoskeleton is impacted by several fundamental changes in AMD [143]. The geometry of the monolayer alters significantly at AMD-affected sites (Figure 3 and Figure 4). These areas are patchy and are more likely to be focal than generally distributed across the posterior pole. Also, these areas seem to be associated with subretinal (subretinal drusenoid deposits = reticular pseudodrusen [144]) and sub-RPE-basal lamina deposits (basal linear deposits and drusen). Oxidative stress in RPE cells leads to the release of free radicals. Peroxidized lipoproteins in drusen and basal linear deposits can initiate and promote inflammation, activated complement cascades, and the upregulation of cytokines and chemokines. As mentioned earlier, these factors external to the RPE could trigger cytoskeleton activation [145]. However, despite these subcellular and molecular changes in the surrounding environment of RPE cells, the RPE seems to be able to react and adapt by re-modeling its geometry, while still maintaining function.

Deposits elevate overlying RPE (drusen) or squeeze underlying RPE (subretinal drusenoid deposit), which results in mechanical stress within RPE cells and affects the cytoskeleton. Also, fluid and/or blood (sub RPE, subretinal) in the course of neovascular AMD can significantly mechanically impact retinal architecture and RPE morphology. In vitro studies found that protein expression linked to cell apoptosis (Bcl2, Bax, and p53) was elevated in RPE cells exposed to mechanical stress [146,147,148]. Continuous mechanical stress can lead to cytoskeleton and RPE phenotype changes [149,150,151,152]. 

Histological studies catalogued 15 different RPE phenotypes associated with non-neovascular and neovascular AMD, with atrophy as the end stage [153,154]. Some phenotypes show a loss of cell contacts and a migration of RPE cells into the neurosensory retina [155,156], where they are seen clinically as hyperreflective foci on SD-OCT. These activities are likely orchestrated by the cell´s cytoskeleton as actin filaments are the main contributors to cell migration in terms of force generation and contraction. In cell culture models, different movement modalities have been described (amoeboid versus mesenchymal), with the main differences in adhesion and contractility characteristics [157,158,159]. Both modalities might be possible for RPE cells in the transition from an epithelial to a mesenchymal phenotype [160]. Still unclear, however, is the initiator for transition and movement: oxygen and nutritional attractant factors in the retina have been discussed, because some cells approach retinal capillaries [155]. Repellent factors in underlying AMD deposits are also a possibility. 

At AMD-affected sites, individual RPE cells lose their normal prism shape, enlarge or fuse, and the local spatial density of RPE cells decreases. In general, RPE cells in AMD-affected areas lose their sharp polygonal appearance and become round or even concave [143]. Further cytoskeleton changes in AMD include the thinning and thickening of F-actin, the splitting and fragmentation of F-actin, and the development of stress fibers (Figure 4 summarizes the cytoskeleton findings in AMD). 

The separation of the cytoskeleton between two adjacent RPE cells may result from mechanical forces from sub-retinal or sub-RPE deposits when cell-to-cell contacts suffer, weaken or disappear under permanent pressure. A similar fragmentation of the F-actin cytoskeleton has been reported in mouse models of different retinopathies or chemically induced RPE degeneration—with basal deposits and a thickened Bruch membrane—as well as in ARPE-19 cell culture models [161,162]. 

Besides maintaining cell shape and geometry, the cytoskeleton is also involved in the traffic and transport of intracellular granules. Alterations in the cytoskeleton have been linked to poor phagocytosis, as shown in stem-cell-derived RPE cells [163], and might indicate abnormality of other cell functions. An indispensable pre-requisite for any cell replacement therapy is to deliver intact and functioning RPE cells.

As recently shown in a histological survey of >29,000 RPE cells [164], a remarkable sign of early AMD in human RPE cells is the re-organization of intracellular autofluorescent lipofuscin granules into aggregates [143], finally leading to a release of these granule aggregates into the sub-RPE space. This re-packing and ejection of granules might be orchestrated by the intracellular cytoskeleton beyond actin, since aggregate-associated changes in actin were not detected in that study.

## 3. Conclusions

The cytoskeleton is an important player in maintaining cell shape and geometry, transmitting signals from the extracellular to the intracellular space (and vice versa), and the intracellular trafficking of granules and proteins. Alterations of the cytoskeleton often result in a change in cell geometry and intracellular trafficking. Age-related changes in the outer retina can be adapted to by the RPE cytoskeleton, while alterations in AMD (splitting, fragmentation, and loss of cytoskeleton) are often accompanied by the occurrence of stress fibers. Because of the importance of polarized pathways for RPE function, these changes to the organizing cytoskeleton will have a major impact on the RPE’s ability to care for choriocapillaris and photoreceptors in aging and AMD. 

In the future, with the advent of new imaging modalities such as adaptive optics assisted scanning laser ophthalmoscopy, in vivo imaging of individual RPE cells might be available. These techniques enable comprehensive studies on RPE geometry in the aging and diseased eye—and thus, might also serve as an indicator of intracellular RPE cytoskeletal health [165,166].
